# Periodontal diseases and coronary artery disease: epidemiological links, pathogenic mechanisms and prospects for comorbidity integrated management

**DOI:** 10.3389/fcimb.2026.1946319

**Published:** 2026-10-06

**Authors:** Chengkui Xu, Tong Yu, Lulu Zhang

**Affiliations:** 1 Department of Cardiology Center, The First Hospital of Jilin University, Changchun, China; 2 First Operating Room, The First Hospital of Jilin University, Changchun, China; 3 Department of Hepatobiliary and Pancreatic Surgery, General Surgery Center, The First Hospital of Jilin University, Changchun, China

**Keywords:** atherosclerosis, cardio-oral comorbidity, coronary artery disease, multidisciplinary management, periodontal disease, periodontal pathogens, systemic inflammation

## Abstract

Periodontal disease (PD) and coronary artery disease (CAD) are two highly prevalent chronic systemic disorders with overlapping population risk profiles. Accumulating observational, cellular, and genetic studies have suggested potential positive correlations between PD severity, periodontal pathogen load, and CAD incidence, coronary lesion severity, and major adverse cardiac events, whereas Mendelian randomization analyses may not support a direct causal link between the two illnesses. Several synergistic pathogenic pathways may mediate their association: periodontal pathogens may translocate into peripheral circulation to colonize atherosclerotic plaques and exert potential direct vascular toxicity; sustained systemic low-grade inflammation may disrupt endothelial homeostasis; molecular mimicry may induce cross-autoimmune vascular impairment; shared susceptibility loci and conventional cardiovascular risk factors may jointly amplify vascular damage. Standard non-surgical periodontal therapy may reduce systemic inflammatory burden and improve endothelial function, and clinical and microbial periodontal markers may serve as non-invasive biomarkers for CAD risk stratification. This review synthesizes epidemiological evidence and multilayered pathogenic mechanisms and proposes bidirectional collaborative interventions and multidisciplinary cardiostomatological management frameworks for comorbid patients. Further large-scale randomized controlled trials and multi-ethnic genetic analyses are still required to clarify causal inference and potential long-term cardiovascular benefits of periodontal intervention.

## 1 Introduction

Periodontal disease (PD) is a chronic infectious inflammatory disorder triggered by a homeostatic imbalance in the dental plaque biofilm, with lesions involving the periodontal supporting tissues, including the gingiva, periodontal ligament, and alveolar bone (Griffiths and Barbour, 2010). Affecting approximately 90% of the global population, PD ranks as the sixth most prevalent human disease worldwide (Pavlic et al., 2021). Clinically, PD is categorized into gingivitis and periodontitis based on the severity of tissue involvement. Gingivitis is an early reversible lesion, characterized by gingival erythema and bleeding on probing (BOP). Standardized supragingival scaling and long-term plaque control can restore periodontal tissue health (Laurent et al., 2007). Without timely intervention, the persistent inflammatory progression of untreated gingivitis leads to irreversible damage to the periodontal ligament and cementum, manifested as clinical attachment loss (CAL), alveolar bone resorption, deepened periodontal pockets, and sustained BOP, and ultimately results in tooth mobility and loss, which is defined as periodontitis (Zulkapli et al., 2026).

Epidemiological data indicate that mild periodontitis affects 45%–50% of adults, while severe periodontitis accounts for approximately 11.2% of the adult population (Maboudi et al., 2024). Among elderly individuals aged 65 years and above, the overall prevalence of periodontitis exceeds 60%, with males carrying a significantly higher disease risk than females (Sanz et al., 2018; Kumar et al., 2025). In clinical practice, non-surgical periodontal therapy (NSPT) serves as the first-line intervention for mild to moderate periodontitis, consisting of core procedures such as supragingival scaling, subgingival curettage, and root planing (Su et al., 2025). For severe periodontitis, auxiliary periodontal surgeries such as flap debridement, gingival grafting, and bone augmentation are required as part of a combined treatment approach (Laurent et al., 2007). The core objective of all periodontal treatments is to eliminate pathogenic biofilm on root surfaces, reduce the load of periodontal pathogens, prevent the invasion of bacteria and their toxic products into the bloodstream through damaged periodontal epithelium, and alleviate local chronic inflammatory responses (Jeffcoat et al., 2014). A growing body of clinical and basic research in recent years has confirmed that PD is not an isolated disease confined to the oral cavity. Its chronic inflammatory state interacts closely with multiple systemic chronic diseases. Coupled with its extremely high global prevalence, comprehensive prevention and control of PD has become an indispensable component in the field of chronic disease management.

Coronary artery disease (CAD) is a systemic inflammatory vascular disorder pathologically centered on coronary atherosclerosis. Progressive proliferation of atherosclerotic plaques narrows the coronary arterial lumen, causing insufficient myocardial blood and oxygen supply and subsequent ischemic myocardial injury (Malakar et al., 2019; Mirnejad et al., 2018). CAD ranks first globally in terms of mortality and disability, contributing to roughly one-third of all deaths among individuals aged over 35 years, with the risk of morbidity and mortality rising markedly with advancing age (Ralapanawa and Sivakanesan, 2021). Atherosclerosis is the fundamental pathological mechanism underlying CAD development and progression: disruption of the vascular endothelial barrier facilitates infiltration and oxidative modification of low-density lipoprotein (LDL) beneath the endothelium, thereby promoting the adhesion of peripheral monocytes and their differentiation into foam cells, thereby forming early fatty streaks. As the disease advances, the proliferation of vascular smooth muscle cells and massive collagen deposition generate fibrous plaques, accompanied by continuous expansion of lipid cores and vascular calcification, ultimately forming vulnerable atherosclerotic plaques. Plaque rupture activates the systemic coagulation cascade to induce thrombosis, which completely occludes coronary arteries and triggers severe adverse outcomes including malignant arrhythmia, sudden cardiac death, and heart failure (Malakar et al., 2019; Shahjehan et al., 2026).

CAD is classified into stable ischemic heart disease and acute coronary syndrome (ACS) according to clinical manifestations and disease acuity. ACS further encompasses three critical subtypes: unstable angina, non-ST-segment elevation myocardial infarction (NSTEMI) and STEMI (Shahjehan et al., 2026). Conventional epidemiological studies have identified advanced age, smoking, hypertension, dyslipidemia, type 2 diabetes mellitus and obesity as classic independent risk factors for CAD. Nevertheless, these risk factors alone cannot fully explain the disparities in CAD incidence across populations, implying the existence of potential pathogenic triggers that remain insufficiently elucidated (Abou-Raya et al., 2002).

Mounting epidemiological and preclinical evidence over recent decades suggests a potential association between PD and CAD. Chronic inflammatory burden from periodontal lesions and sustained exposure to periodontal pathogens may participate in the progression of coronary atherosclerosis and elevate the risk of acute adverse cardiovascular events. However, conclusions from existing studies remain inconsistent: observational cohort studies and basic experiments consistently confirm a correlation between the two conditions, whereas several genetic causal inference studies using Mendelian randomization(MR) fail to establish a direct causal relationship between PD and CAD. In light of such contradictory findings, this paper synthesizes multi-type research including cross-sectional, case-control, long-term interventional cohort, and genetic association studies, summarizes epidemiological correlations and mediating mechanisms linking PD and CAD, explores the clinical value and translational limitations of integrated comorbidity management for the two diseases, and provides references for multidisciplinary collaborative diagnosis and treatment of chronic diseases involving cardiology and stomatology departments.

## 2 Literature retrieval and screening procedures

To systematically collate and interpret existing research exploring the potential correlation and multilayered pathogenic links between PD and CAD, comprehensive literature searches were independently performed across PubMed and Web of Science Core Collection. Literature covering all available years from the establishment of each database through the date of manuscript submission was captured, with no upper or lower limits set on publication timepoints. We constructed compound search formulas by combining standardized Medical Subject Headings (MeSH) and corresponding free-text keywords. The core retrieval terms covered periodontal disease, periodontitis, key periodontal pathogens, coronary atherosclerosis, coronary artery disease, systemic inflammation, endothelial injury, autoimmunity, and shared genetic susceptibility loci, which fully covered the epidemiological, cellular, molecular, and genetic dimensions discussed in this review.

All retrieved literature underwent multi-round screening based on predefined inclusion and exclusion standards. Eligible study types included human observational research (cross-sectional, case-control, and prospective cohort studies), periodontal interventional follow-up trials, *in vitro* cellular mechanistic experiments, and Mendelian randomization-based genetic causal inference studies. Both systematic reviews and narrative reviews were reserved to assist supplementary literature mining and cross-verification of inconsistent research conclusions in existing original articles. Excluded literature types included conference abstracts without complete data, isolated single case reports, duplicate publications repeatedly released by the same research team, and articles that failed to provide complete periodontal indicators or cardiovascular endpoint data for quantitative correlation analysis.

After removing duplicate entries using the built-in deduplication functions of the two databases, two authors separately completed preliminary screening by reading titles and abstracts. Potentially relevant articles that could not be judged simply by abstract information were subjected to full-text reading for secondary eligibility assessment. Any disagreements arising during the screening process were resolved through group discussion among all authors to unify judgement criteria. Ultimately, all qualified studies were integrated and sorted to summarize consistent and contradictory epidemiological evidence, unpack multi-interconnected pathogenic pathways, and further elaborate integrated multidisciplinary management strategies for patients co-affected by PD and CAD.

## 3 Epidemiological evidence linking PD and CAD

A spectrum of epidemiological studies has explored the association between PD and CAD from multiple dimensions, including cross-sectional, case-control, long-term prospective cohort, *in vitro* cellular, and genetic causal inference designs. Most observational studies consistently demonstrate that PD prevalence, severity of periodontal tissue destruction, and periodontal pathogen load are positively correlated with CAD incidence, severity of coronary lesions, and risk of acute adverse cardiovascular events. Nevertheless, genetic causal inference studies using MR and a small number of clinical case-control investigations fail to establish a direct causal effect between the two diseases, suggesting complex confounding factors and ongoing controversy regarding their potential causal relationship.

### 3.1 Evidence from cross-sectional studies

Numerous single-center cross-sectional studies based on coronary angiography and population surveys across multiple countries have shown that periodontal inflammation, tooth loss, and subgingival pathogen colonization are independent risk factors for CAD, and that the extent of periodontal damage correlates with risk factors such as the number of stenotic coronary vessels and multivessel coronary artery disease.

A 2009 Japanese study enrolling 174 subjects undergoing coronary angiography revealed that an elevated comprehensive periodontal risk score significantly increased the odds of CAD after adjustment for confounders (OR = 2.317, P = 0.0171), with this association more prominent in participants under 60 years old with normal glucose tolerance (Oe et al., 2009). A subsequent Brazilian cross-sectional study further found that retaining fewer than 20 natural teeth and self-reported poor oral health were significantly associated with high atherosclerotic burden (PR = 1.09, P = 0.02; PR = 1.22, P = 0.03) (Gomes et al., 2012). Supplementing evidence linking periodontal destruction to coronary lesions from the perspective of tooth loss. A cohort of 106 participants in Croatia demonstrated that the prevalence of chronic periodontitis was markedly higher in patients with ACS and stable CAD compared with the control group without CAD (P<0.001). Moreover, the proportion of patients with chronic periodontitis rose alongside increasing coronary lesion severity (Vražić et al., 2015). Directly illustrating a parallel increase in coronary lesion severity and periodontal inflammatory burden. A 2019 Korean study of 88 subjects with suspected CAD reported that tooth loss of ≥10 teeth was an independent risk factor for obstructive CAD after adjustment for confounders (OR = 8.02, P = 0.006) (Lee et al., 2019). This finding was corroborated by a 2011 Finnish coronary angiography cohort, which similarly observed substantially elevated risks of stable CAD and ACS among individuals with 8–17 missing teeth and ≥7 periodontal pockets with probing pocket depth (PPD) ≥6 mm (Buhlin et al., 2011). Both studies identified a positive correlation between the number of missing teeth and the count of stenotic coronary vessels, suggesting that more severe periodontal tissue destruction corresponds to a higher risk of multivessel coronary stenosis. A 2013 Chinese study yielded consistent results. The study recruited 115 non-smoking, non-diabetic patients aged ≥60 years with angiographically confirmed coronary stenosis ≥50%. The Gensini score, which quantifies coronary stenosis severity, was significantly higher in the severe periodontitis group than in the periodontally healthy group (P = 0.006). After adjustment for confounders, PPD and plaque index (PLI) were each independently and positively correlated with the severity of coronary stenosis (Yang et al., 2013). Indicating that greater periodontal inflammation is associated with more severe coronary luminal narrowing in elderly populations. A 2020 Turkish study of 309 patients undergoing coronary angiography supplemented stratified data, demonstrating that periodontitis and edentulism were both independent CAD risk factors (OR = 2.48, P = 0.010; OR = 2.01, P = 0.040), and that these patients were more likely to develop multivessel stenosis (P = 0.003) (Bilgin Çetin et al., 2020). A recent single-center prospective cross-sectional study conducted in Romania in 2026 consecutively enrolled 200 hospitalized patients with acute myocardial infarction (AMI). The analysis detected a direct positive correlation between the number of missing teeth and the number of stenotic coronary arteries (Pearson’s r = 0.19, p = 0.007), and tooth loss exceeding 20 teeth was significantly associated with multivessel CAD (OR = 1.84, p = 0.047) (Cinezan et al., 2026). A 2026 retrospective cross-sectional study of 472 hospitalized Chinese patients further quantified the magnitude of the risk. After adjustment for confounding, each 1-mm increase in PPD was associated with a 25% increase in the odds of CAD (OR = 1.25, P = 0.003) (Lu and Lu, 2026).

Cross-sectional microbiological evidence establishes a direct causal link between oral pathogenic bacteria and coronary lesions. A 2021 multicenter Serbian study performed PCR testing on atherosclerotic plaques harvested from patients undergoing coronary artery bypass grafting. Homologous periodontal pathogens including Porphyromonas gingivalis (Pg) and Aggregatibacter actinomycetemcomitans (Aa) were detected in both coronary plaques and subgingival plaque samples collected from the same patients, and PPD values were significantly higher among individuals with pathogen-positive coronary plaques (Pavlic et al., 2021). A 2023 Chinese cross-sectional case-control study of 382 AMI patients utilized quantitative qPCR to detect circulating Pg nucleic acids, confirming that bloodstream colonization by this pathogen was independently associated with AMI (OR = 1.853, P<0.05) and exacerbated coronary stenosis severity (Wu et al., 2023).

Collectively, cross-sectional evidence across multi-ethnic cohorts consistently establishes a graded positive correlation between periodontal destruction severity and coronary atherosclerotic burden. Clinical periodontal indicators, including probing pocket depth, clinical attachment loss, and tooth loss, serve as quantifiable markers of cumulative oral inflammatory exposure. At the same time, microbial detection data provide direct biological evidence that oral periodontal pathogens can enter the systemic circulation and colonize coronary lesions. From a translational perspective, these cross-sectional observations support the feasibility of incorporating routine oral examination into cardiovascular risk screening for hospitalized patients with CAD. Nevertheless, inherent limitations of cross-sectional design preclude causal inference. All associations are captured at a single time point, which cannot distinguish whether periodontal inflammation precedes coronary lesion formation or arises as a secondary complication of systemic cardiovascular metabolic dysfunction.

### 3.2 Evidence from case-control studies

Multiple matched case-control studies uniformly confirm that moderate to severe periodontitis represents an independent risk factor for first-ever myocardial infarction and ACS. In addition, periodontal inflammatory indices can concurrently reflect the severity of myocardial injury and ventricular load.

A 2016 PAROKRANK multicenter matched case-control study in Sweden, enrolled 805 1:1 matched pairs of patients with first myocardial infarction and healthy controls. After adjustment for confounders, periodontitis was independently associated with incident myocardial infarction (OR = 1.28, 95% CI, 1.03–1.60). The proportion of patients with severe periodontitis in the case group was 2.5 times that observed in controls (Rydén et al., 2016). Indicating a linear elevation in acute coronary event risk with worsening periodontal disease severity. A 2021 single-center Polish study recruited 71 patients with AMI and 40 healthy controls. Significantly elevated BOP and approximal plaque index (API) values were observed in individuals with myocardial infarction. Each 1% increase in API raised the risk of myocardial infarction. Furthermore, BoP and API levels predicted cardiac troponin I concentrations, while CAL correlated with elevated BNP levels (Wojtkowska et al., 2021). These findings demonstrate that PD not only increases the incidence of AMI but also exacerbates myocardial necrosis and ventricular overload injury. A 2025 retrospective stratified case-control analysis of 166 ACS patients in Romania further validated these conclusions. Patients comorbid with periodontitis exhibited a 2.294-fold higher risk of STEMI (OR = 2.294, P = 0.036), alongside significantly higher rates of left main coronary artery disease and triple-vessel severe coronary stenosis (P<0.001) (Rus et al., 2025).

Matched case-control designs eliminate partial demographic confounding via pairwise matching, and their pooled findings robustly confirm moderate-to-severe periodontitis as an independent risk factor for initial acute coronary events, including myocardial infarction and STEMI. Notably, periodontal inflammatory biomarkers such as BOP and API are not only correlated with CAD incidence but also linearly associated with myocardial injury markers and ventricular overload indicators, suggesting that periodontal infectious inflammation may amplify the severity of cardiac tissue damage following acute coronary syndrome. Despite these consistent positive associations, case-control studies remain vulnerable to recall bias: patients with severe cardiovascular disease may tend to overreport poor oral health status, which may artificially magnify the observed association strength between PD and CAD.

### 3.3 Evidence from prospective cohort and interventional follow-up studies

Unintervened large prospective cohorts confirm that baseline periodontitis independently elevates the risk of incident CAD and long-term major adverse cardiac events (MACE). A 2024 cohort study drawing on UK Biobank data followed 480,000 participants for a median of 13.8 years. Cox regression analysis revealed a significantly higher risk of new-onset CAD among individuals with PD (HR = 1.09, P<0.001). Each standard deviation increase in the PD polygenic risk score (PRS) corresponded to a synchronous increase in CAD susceptibility (OR = 1.033, P<0.0001) (Sanghvi et al., 2024). A 2022 Japanese follow-up cohort of 77 non-smoking patients post-percutaneous coronary intervention (PCI) demonstrated a median MACE incidence of 26.5% in the periodontitis group, versus merely 7.1% in the periodontally healthy group, accompanied by markedly reduced MACE-free survival in patients with PD (P = 0.034). Rates of non-target vessel revascularization and Pg detection were also significantly higher among patients with periodontitis (Fukushima et al., 2022).

Periodontal interventional follow-up studies directly validate that periodontal treatment can reverse vascular damage and reduce the risk of adverse coronary events. A 2009 Japanese interventional trial of 101 non-smoking CAD patients found severely impaired endothelium-dependent vasodilation in subjects complicated by chronic periodontitis (P<0.001). NSPT administered over 24 weeks produced substantial improvements in endothelial vasodilatory function (Higashi et al., 2009). Preliminarily verifying the reversibility of endothelial injury mediated by chronic periodontal inflammation. A 2019 Brazilian study followed 206 dialysis patients awaiting kidney transplantation who received systematic periodontal therapy and 203 untreated controls for 24 months. Kaplan-Meier survival analysis demonstrated superior coronary event-free survival in the periodontal intervention group (Log-rank P = 0.009). After adjustment for confounders, periodontal treatment independently reduced the risk of adverse coronary events (HR = 0.31, P = 0.020) (Santos-Paul et al., 2019). Indicating that long-term periodontal maintenance reduces atherosclerotic cardiovascular endpoints.

Long-term prospective cohort data overcome the temporal limitations of cross-sectional and case-control research by recording baseline periodontal status before CAD onset, providing high-level observational evidence that untreated periodontitis elevates long-term MACE risk independent of traditional cardiovascular confounders. More critically, interventional follow-up trials provide translational evidence of a modifiable association: standardized non-surgical periodontal therapy reverses endothelial dysfunction and reduces systemic inflammatory load, forming the core clinical rationale for combined cardio-stomatological intervention. A key limitation of existing intervention trials is their relatively short follow-up duration, which cannot capture the long-term protective cardiovascular effects of sustained periodontal maintenance over decades.

### 3.4 Supporting evidence from *in vitro* cellular experiments and genetic association studies

*In vitro* cellular experiments recapitulate the pathological processes through which periodontal pathogenic factors drive coronary atherosclerosis at the cellular and molecular levels. A 2014 Thai *in vitro* study found that lipopolysaccharide(LPS) derived from Pg (Pg-LPS), when combined with cholesterol crystals, activates the NLRP3 inflammasome in macrophages and coronary artery endothelial cells, triggering secretion of mature IL-1β and initiating early inflammatory responses characteristic of atherosclerosis (Champaiboon et al., 2014). A 2018 Colombian study showed that LPS from Eikenella corrodens (Ec-LPS) binds to the TLR4 receptor on coronary endothelial cells, activating the ERK1/2 and NF-κB signaling cascades. This stimulates the robust release of proatherogenic chemokines from the coronary endothelium and upregulates membrane-bound ICAM-1, thereby enhancing monocyte adhesion and recapitulating early endothelial lesions (Viafara-Garcia et al., 2018). Additional cellular research has established that Pg infection suppresses the endothelial protective nitric oxide synthases eNOS and nNOS while inducing the expression of the injury-inducible iNOS, thereby disrupting endothelial nitric oxide (NO) homeostasis (Sampath et al., 2021).

Genetic association studies confirm shared susceptibility genes and regulatory pathways between PD and CAD. A 2015 multicountry genome-wide analysis across European cohorts identified three categories of shared susceptibility loci for both diseases: ANRIL, CAMTA1/VAMP3, and the plasminogen gene (PLG). All shared genes are regulated by the transforming growth factor-β (TGF-β) signaling pathway, providing a genetic basis for the high prevalence of comorbidity between the two conditions (Schaefer et al., 2015). A 2024 functional validation study further demonstrated that variants at the ANRIL locus simultaneously increase the genetic risk of developing PD and CAD, adding supplementary genetic evidence for their association (Shi et al., 2024).

*In vitro* cellular assays dissect the molecular cascade through which periodontal pathogens trigger endothelial injury, lipid dysregulation, and vascular calcification, offering mechanistic explanations for the epidemiological correlations observed in human population studies. Genetic association analyses further identify shared susceptibility loci regulated by TGF-β signaling, thereby explaining interindividual heterogeneity in the strength of PD-CAD association: carriers of risk genotypes exhibit greater susceptibility to vascular damage under identical periodontal inflammatory burden. These genetic and preclinical data establish a framework of biological plausibility linking oral infection to atherosclerosis. Yet, such evidence cannot validate population-level causality, as cell- and genetic-based studies fail to account for complex real-world confounding factors such as lifestyle and metabolic comorbidities.

### 3.5 Negative and contradictory studies

The cross-sectional, case-control, cohort, and basic science studies outlined above generally support a positive association between PD and CAD. However, several genetic causal inference studies using MR and a small subset of clinical case-control investigations fail to detect an independent correlation between the two disorders, generating contradictory evidence. These discrepancies suggest that shared risk factors may confound associations observed in observational studies, and PD cannot yet be definitively classified as a direct causal factor for CAD.

MR analyses use periodontitis genetic loci as instrumental variables to control for confounding by factors such as smoking and diabetes. Yet, most such studies fail to validate a direct genetic causal link between PD and CAD. A 2020 two-sample MR study from the UK leveraged genome-wide association study (GWAS) genetic datasets and selected five periodontitis-related single nucleotide polymorphisms (SNPs) as instrumental variables. Primary inverse variance weighting and multiple sensitivity analyses yielded no evidence of a causal relationship between genetic susceptibility to periodontitis and CAD (Bell et al., 2020). A 2021 Chinese two-sample MR study replicated these null findings using GWAS data, selecting 47 independent genetic loci associated with periodontitis and dental caries as instrumental variables, and found no causal association between periodontitis and CAD (Zhou et al., 2021). A 2024 bidirectional two-sample MR study from China repeated the validation using GWAS data; neither forward nor reverse causal inference produced statistically significant positive results, refuting the hypothesis that periodontitis directly induces coronary atherosclerosis (Li et al., 2024).

Negative results have also been reported in clinical case-control research. A 2024 single-center Iranian case-control study enrolled 50 patients with CAD and 50 healthy controls. After adjustment for confounders, the prevalence of periodontitis, PPD, CAL, BOP, and subgingival Pg colonization exhibited no independent statistical correlation with CAD. While poorer overall periodontal health was superficially observed among CAD patients, an independent association between the two diseases could not be confirmed (Maboudi et al., 2024).

### 3.6 Section summary

Cross-sectional, case-control, prospective population cohort, *in vitro* cellular, and genetic susceptibility association studies reach highly consistent conclusions, collectively demonstrating that PD status, periodontal inflammatory burden, and distant colonization by periodontal pathogens are significantly correlated with CAD incidence, severity of coronary lesions, and long-term risk of acute cardiovascular events (**Table 1**). Systematic periodontal intervention ameliorates vascular endothelial function and reduces the incidence of long-term adverse coronary events. Microbial testing can detect homologous periodontal pathogens in atherosclerotic plaques and peripheral blood. At the same time, molecular and genetic experiments have clarified some potential signaling pathways and genetic susceptibility foundations underlying this association. Each category of research design delivers distinct translational value: cross-sectional surveys support routine oral screening for hospitalized CAD patients; matched case-control data links periodontal inflammatory markers to the severity of acute cardiac injury; long-term prospective cohorts establish the temporal sequence between baseline periodontal damage and subsequent coronary events; interventional trials validate the reversibility of inflammation-mediated vascular dysfunction, while cellular and genetic experiments elaborate the biological plausibility of oral-cardiac crosstalk.

**Table 1:** Summary of representative studies on PD and CAD.

Authors (year/country)	Study type	Study subjects	Sample size	Periodontal assessment indicators	CAD diagnostic criteria	Adjusted confounders	Core effect size and p-value	Main conclusions	Reference
Oe et al. (2009/Japan)	Single-center cross-sectional clinical observational study	Hospitalized patients undergoing simultaneous coronary angiography and periodontal examination	Total 174 cases: 99 in CAD group, 75 in non-CAD group	Comprehensive periodontal risk score constructed from 5 indicators	Coronary angiography confirmed coronary stenosis >50%, or history of PCI/CABG defined as CAD; no coronary stenosis defined as non-CAD	Hypertension, glucose tolerance status, statin use	Multivariate OR = 2.317, 95%CI 1.108–3.315, P = 0.0171; significant difference in periodontal scores only in subjects <60 years old with normal glucose tolerance (P<0.05)	Comprehensive periodontal risk score is an independent risk factor for CAD, and this association only exists in young and middle-aged subjects with normal glucose tolerance	(Oe et al., 2009)
Yang et al. (2013/China)	Single-center retrospective cross-sectional study	Elderly CHD patients aged ≥60 years without smoking or diabetes, divided into 4 groups by periodontitis severity	Initial screening 853 cases, final inclusion 115 cases	Armitage classification: CAL, PPD, Bleeding Index (BI), Calculus Index (CI), Plaque Index (PLI)	Judkins method coronary angiography, Gensini score to quantify coronary stenosis severity	Mutual adjustment for all periodontal indicators	Regression coefficient of PD = 0.62, P = 0.002; regression coefficient of PLI = 0.57, P = 0.024; Gensini score was significantly higher in severe periodontitis group (P = 0.006)	In elderly patients (aged >60 years) with CHD, the severity of periodontitis is positively correlated with the severity of coronary stenosis; PD and PLI are independent periodontal indicators associated with coronary lesions	(Yang et al., 2013)
Pavlic et al. (2021/Serbia)	Microbiological cross-sectional study	Patients with carotid/CA undergoing endarterectomy/bypass surgery	Total 58 cases: 30 in carotid group, 28 in coronary group	Plaque Index (PI), Gingival Index (GI), Sulcus Bleeding Index (SBI), PPD; PCR detection of 5 periodontal pathogens	Clinically confirmed CA requiring CABG	No multivariate regression adjustment, only consistency test and rank sum test	Kappa value for Tannerella forsythia in carotid artery=0.67, P<0.001; consistency of Pg, Aa, Td in coronary artery all P<0.001	Homologous periodontal pathogens can be detected in coronary atherosclerotic plaques, and periodontal infection is involved in the progression of atherosclerosis	(Pavlic et al., 2021)
Wu et al. (2023/China)	Single-center case-control cross-sectional study	AMI cases aged <75 years	Total 460 cases: 382 in AMI group, 78 in control group	qPCR detection of Pg in oral cavity, cell-free DNA (cfDNA) and genomic DNA (gDNA)	In accordance with the 4th global definition of myocardial infarction, SYNTAX score was used to evaluate coronary lesion severity	Age, gender, smoking, diabetes, dyslipidemia, homocysteine	Adjusted OR of peripheral blood gDNA Pg for predicting AMI = 1.853, 95%CI 1.042–3.295, P<0.05	Only Pg invading the blood circulation is an independent risk factor for AMI, which can aggravate coronary stenosis and increase systemic inflammation	(Wu et al., 2023)
Lu et al. (2026/China)	Single-center retrospective observational cohort study	Hospitalized adults aged ≥18 years	Total 472 cases: 289 in periodontitis group, 183 in control group	2017 periodontal staging and grading criteria, detecting PPD, CAL, BOP, alveolar bone loss	Composite cardiovascular endpoint: CHD, myocardial infarction, stroke, heart failure	Age, gender, smoking and alcohol consumption, diabetes, hypertension, BMI	Adjusted OR for overall periodontitis=1.87, 95%CI 1.28–2.72, P = 0.001; OR for stage III/IV periodontitis=2.41, P<0.002; OR for grade C progressive periodontitis=2.28, P = 0.002	Periodontitis is an independent risk factor for CHD; the more severe the periodontal lesion, the higher the risk of CHD	(Lu and Lu, 2026)
Cinezan et al. (2026/Romania)	Prospective cross-sectional study	Hospitalized patients with AMI	Total 200 cases	The total number of missing permanent teeth	Judkins method coronary angiography, the number of major coronary arteries with severe stenosis was used to define the severity of CAD	Age, gender, smoking, diabetes, obesity, dyslipidemia, hypertension	After multivariate adjustment, OR of >20 missing teeth for predicting multi-vessel CAD = 1.84, 95%CI 1.01–3.34, P = 0.047	A large number of missing teeth is an independent risk factor for multi-vessel CAD in patients with AMI	(Cinezan et al., 2026)
Rydén et al. (2016/Sweden)	Multi-center matched case-control study	Patients with first myocardial infarction	Total 1610 cases: 805 cases, 805 controls	Panoramic radiograph measurement of residual alveolar bone ratio of tooth roots, divided into healthy/mild-moderate/severe periodontitis groups	In accordance with the international diagnostic criteria for AMI, first myocardial infarction was confirmed as the CHD endpoint	Diabetes, smoking history, education level, marital status	Unadjusted OR = 1.49, 95%CI 1.21–1.83, P<0.001; Multivariate adjusted OR = 1.28, 95%CI 1.03–1.60, P<0.05	Periodontitis is an independent risk factor for first myocardial infarction, and the association remains significant after adjusting for traditional confounding factors	(Rydén et al., 2016)
Wojtkowska et al. (2021/Poland)	Single-center case-control cross-sectional study	Hospitalized patients with AMI aged <65 years	Total 111 cases: 71 cases, 40 controls	Number of remaining teeth, Approximal Plaque Index (API), BOP, PPD, bleeding periodontal pockets ≥4mm CAL	Coronary angiography confirmed AMI (STEMI/NSTEMI), coronary lesions divided into single-vessel/multi-vessel	Age, gender, smoking, diabetes, lipid-lowering/antiplatelet drugs, metabolic syndrome indicators	Logistic regression adjusted OR of API = 1.08, P<0.001; OR of BOP = 0.93, P = 0.001; For every 1mm increase in CAL, BNP increased by 31.33pg/ml, P = 0.003	API is an independent risk factor for myocardial infarction, and periodontal inflammatory indicators are correlated with systemic inflammation and heart failure markers	(Wojtkowska et al., 2021)
Rus et al. (2025/Romania)	Single-center retrospective observational cohort study	Hospitalized patients with ACS	Total 166 cases: 103 with periodontitis, 63 without periodontitis	Based on medical records: gingival bleeding, gingival recession, halitosis, alveolar bone loss, tooth loosening, previous periodontal treatment history	Coronary angiography confirmed CHD, divided into single-vessel, double-vessel, three-vessel/left main lesions; unstable angina, NSTEMI, STEMI distinguished by electrocardiogram and myocardial enzymes	Gender, smoking, systemic metabolic/immune diseases, heart failure	After multivariate adjustment, OR of periodontitis for predicting STEMI = 2.294, 95%CI 1.056–4.983, P = 0.036; The proportion of three-vessel coronary lesions, heart failure and arrhythmia complications in periodontitis group was significantly increased (all P<0.001)	Periodontitis is an independent risk factor for STEMI; ACS patients with periodontitis have more severe coronary lesions and higher risk of cardiovascular complications	(Rus et al., 2025)
Higashi et al. (2009/Japan)	Single-center randomized controlled intervention study	Patients with confirmed CAD	Total 101 cases: 48 with periodontitis (24 treated/24 untreated), 53 without periodontitis	Periodontal pocket depth, CAL, proportion of bleeding sites on probing	Coronary angiography confirmed proximal coronary stenosis ≥70%, or single-vessel stenosis 80% with typical angina	Smoking population excluded at enrollment; baseline matched for blood pressure, lipids, diabetes, coronary lesions and medication	After 24 weeks of periodontal treatment: hs-CRP and IL-6 decreased (P<0.05), and forearm diastolic blood flow increased significantly (P<0.001); After L-NMMA blocked NO, the endothelial difference between groups disappeared completely	Periodontitis aggravates endothelial dysfunction in patients with CHD by reducing NO availability; standardized periodontal treatment can reduce systemic inflammation and repair endothelium-dependent vasodilation	(Higashi et al., 2009)
Santos-Paul et al. (2019/Brazil)	Single-center retrospective historical control cohort study	Maintenance hemodialysis patients waiting for kidney transplantation	206 cases in intervention group, 203 cases in historical control group, total 409 cases	Panoramic radiograph + full-mouth six-site probing, measuring gingival recession, PPD, CAL	Coronary angiography confirmed coronary stenosis ≥70% in single vessel or ≥50% in left main trunk defined as CAD	Age, gender, dyslipidemia, smoking, diabetes, previous coronary intervention, dialysis duration	1. Prevalence of CAD in severe periodontitis patients 83.3%, P = 0.02; Multivariate Cox: periodontal treatment reduced composite cardiovascular events HR = 0.43, 95%CI 0.22–0.87, P = 0.019	Moderate to severe periodontitis is significantly associated with CAD in dialysis patients; standardized periodontal treatment can independently reduce the risk of coronary events and cardiovascular death within 24 months	(Santos-Paul et al., 2019)
Fukushima et al. (2022/Japan)	Single-center retrospective cohort study	Non-smoking patients with primary coronary lesions undergoing PCI with DES implantation	Total 77 cases: 49 in periodontitis group, 28 in non-periodontitis group	Community Periodontal Index (CPI),PPD, CAL, BOP	Coronary angiography confirmed primary coronary stenosis lesions, drug-eluting stents implanted	Baseline age, chronic diseases, lipids, coronary lesions and medication were balanced between the two groups, no multivariate regression; only Log-rank survival test	After a median follow-up of 1635 days, the incidence of MACE in periodontitis group was 26.5% vs 7.1% in control group, Log-rank P = 0.034; Non-target vessel revascularization in periodontitis group 18.4% vs 0% in control group, P = 0.015	In non-smoking CHD patients undergoing PCI, baseline periodontitis significantly increases the risk of long-term major adverse cardiac events	(Fukushima et al., 2022)
Sanghvi et al. (2024/UK)	large-scale prospective longitudinal cohort study	General community adults aged 40–69 in UK Biobank	Total 481,915 cases: 91,022 with PD, 390,893 without PD	Baseline self-reported questionnaire: PD defined as any of gingival pain, gingival bleeding, tooth loosening	ICD-10 codes I20~I25 defined new-onset CAD, confirmed by hospital admission records and death registration	Age, ethnicity, blood pressure, BMI, smoking, glycosylated hemoglobin, LDL, hs-CRP, social deprivation index	Cox regression: HR of periodontal disease for new-onset CAD = 1.09, 95%CI 1.07–1.13, P<0.001; For every 1 SD increase in periodontal PRS, OR of CAD = 1.033, 95%CI 1.021–1.046, P<0.0001	Multi-dimensional evidence confirms that periodontal disease independently increases the risk of new-onset CHD and induces subclinical myocardial hypertrophic remodeling	(Sanghvi et al., 2024)
Champaiboon et al. (2014/Thailand)	*In vitro* cell mechanistic study	Human primary M1/M2 macrophages, Human Coronary Artery Endothelial Cells (HCAEC)	NA	*In vitro* stimulant: Pg-LPS	*In vitro* simulation of coronary endothelial pathological cells	NA	After Pg-LPS+ATP stimulated macrophages, IL-1β increased significantly, P<0.05; Pg-LPS+cholesterol crystals could induce M2 macrophage IL-1β in a dose-dependent manner, P = 0.029	Pg-LPS can synergize with cholesterol crystals to activate macrophage NLRP3 inflammasome and release a large amount of pro-inflammatory IL-1β	(Champaiboon et al., 2014)
Viafara-Garcia et al. (2018/Colombia)	*In vitro* cell mechanistic study	HCAEC	NA	*In vitro* stimulant: Ec-LPS	*In vitro* simulation of coronary endothelial pathological cells	NA	After Ec-LPS stimulation, IL-8, MCP-1, GM-CSF increased significantly, P<0.05; TLR4 neutralizing antibody significantly inhibited the secretion of all pro-inflammatory factors (P<0.05)	Ec-LPS activates coronary endothelial cells through TLR4/ERK/NF-κB pathway, releases pro-atherosclerotic inflammatory factors, and enhances monocyte adhesion	(Viafara-Garcia et al., 2018)
Bell et al. (2020/UK)	Two-sample Mendelian Randomization Genetic Causal Study	GWAS summary genetic data	Stroke: 44,221 cases/739,957 controls; CAD: 122,733 cases/424,528 controls; cIMT subclinical atherosclerosis 22,179 people	5 GWAS-validated periodontitis susceptibility SNPs were used as genetic instrumental variables	CAD diagnosed by ICD codes, Carotid Intima-Media Thickness (cIMT) was used to assess subclinical atherosclerosis	Genetic analysis automatically adjusted for population genetic principal components, multiple MR models controlled for pleiotropy and abnormal loci	IVW: OR of periodontitis doubling risk for CAD = 1.01, 95%CI 0.99–1.03, P = 0.73; Ischemic stroke OR = 1.00, P = 0.53; Subclinical atherosclerosis β=-0.002, P>0.05	No reliable evidence was found at the genetic level to support a causal relationship between periodontitis and ischemic stroke or CAD	(Bell et al., 2020)
Zhou et al. (2021/China)	Two-sample Mendelian Randomization Genetic Causal Study	GWAS summary genetic data	PD 487,823 people; CAD 184,305 cases/controls	47 genome-wide significant SNPs were used as genetic instrumental variables for PD	CAD confirmed by coronary angiography/medical records	Genetic analysis adjusted for population genetic principal components; Bonferroni correction for multiple testing with P threshold 0.006	In IVW model, there was no causal association between periodontal disease genetic susceptibility and CAD (P>0.05); no statistically significant positive results in all sensitivity analyses	Genetic evidence does not support periodontal disease as a direct cause of CAD, stroke, atrial fibrillation, or heart failure	(Zhou et al., 2021)
Li et al. (2024/China)	Bidirectional Two-sample Mendelian Randomization Genetic Study	GWAS summary genetic data	PD 198,441 cases; CA 61,194 cases	Genome-wide significant threshold SNPs were used as genetic instrumental variables for PD	CA diagnosed by ICD criteria	Genetic principal component adjustment, Bonferroni correction for multiple testing	CA → chronic periodontitis: OR = 2.110, 95%CI 0.208–21.317, P = 0.527; CA → acute periodontitis: OR = 0.414, 95%CI 0.051–3.384, P = 0.644; PD → CA: OR = 1.000, 95%CI 0.999–1.001, P = 0.953	No bidirectional causal association between PD and CA was confirmed at the genetic level	(Li et al., 2024)
Maboudi et al. (2024/Iran)	Single-center Case-Control Study	Patients with CAD confirmed by coronary angiography	Total 100 cases; 50 cases in CAD group, 50 cases in control group	PPD, CAL,BOP; RT-PCR quantitative detection of subgingival Pg load	Coronary angiography with coronary stenosis lesions defined as CAD	Age, gender, periodontal indicators, Pg infection status	Pg positive in CAD group 32% vs 22% in control group, P = 0.260; 2. Multivariate regression: OR of Pg=2.131, P = 0.168; OR of periodontitis=7.089, P = 0.137	This study did not confirm a significant association between subgingival Pg infection and CAD	(Maboudi et al., 2024)

Aa, Aggregatibacter actinomycetemcomitans; AMI, acute myocardial infarction; BOP, bleeding on probing; CAD, coronary artery disease; CAL, clinical attachment loss; CABG, coronary artery bypass grafting; CHD, coronary heart disease; Ec, Eikenella corrodens; GWAS, genome-wide association study; LPS, lipopolysaccharide; PCI, percutaneous coronary intervention; PD, periodontal disease; Pg, Porphyromonas gingivalis; PPD, periodontal pocket depth; Td, Treponema denticola.

Nevertheless, these disparate study designs yield evidence with markedly different inferential strength, which warrants careful distinction when interpreting the PD-CAD relationship. Observational investigations (cross-sectional, case-control, and prospective cohort studies) can establish epidemiological correlation and temporal ordering, but cannot independently verify causal relationships. *In vitro* cellular experiments support the biological plausibility of putative pathogenic cascades, but cannot confirm that these mechanistic pathways operate *in vivo* in human subjects. Existing periodontal interventional follow-up trials show that periodontal treatment improves surrogate endpoints, including endothelial function and systemic inflammatory burden, among individuals with pre-existing CAD; however, these trials are not designed to test whether PD causally triggers the de-novo onset of CAD. MR is the primary analytical approach for population-level causal inference on whether periodontitis susceptibility directly drives CAD risk, and currently available MR analyses have consistently reported null findings; a small subset of clinical case-control studies similarly failed to identify PD as an independent, direct causal factor for CAD.

This persistent divergence between positive observational/preclinical evidence and negative MR/case-control results stems from five systematic methodological and biological heterogeneities, including substantial heterogeneity in operational definitions of both PD (exposure) and CAD (outcome) across primary studies, which collectively explain the literature’s inconsistent conclusions. First, causal identification frameworks differ fundamentally: cross-sectional, case-control, and cohort analyses only quantify epidemiological correlation and cannot fully eliminate residual confounding from shared lifestyle risk factors, including smoking, diabetes, obesity, and high-sugar diets; even multivariate adjustment cannot capture unmeasured socioeconomic, inflammatory, or genetic confounders, which may artificially inflate the observed PD-CAD association. By comparison, MR uses birth-fixed genetic variants as instrumental variables to mitigate reverse causality and confounding bias. Notably, primary studies use highly variable metrics to define periodontal disease exposure. Some investigations use tooth loss with different cut-off values, while others use continuous clinical indices such as PPD or CAL.

Meanwhile, other work relies on binary periodontitis diagnostic criteria or microbial detection of periodontal pathogens. These markers capture distinct dimensions of periodontal destruction and may reflect divergent timelines of cumulative inflammatory exposure, and cannot be treated as equivalent surrogates for PD burden. Similarly, CAD outcomes are not uniformly defined across datasets, ranging from continuous angiographic stenosis scores and specific acute coronary event subtypes to broad registry-based diagnostic codes and composite endpoints merging coronary heart disease, stroke, and heart failure. Research addressing PD-associated endothelial dysfunction among patients with established CAD answers fundamentally different scientific questions from studies evaluating PD as a risk factor for incident STEMI. Such definitional heterogeneity represents an independent source of discrepancy between observational and MR analyses. Most MR studies use broad genetic instruments for periodontitis and generalized CAD diagnostic codes as outcomes.

In contrast, many positive observational analyses use specific periodontal markers (e.g., tooth loss, detection of circulating periodontal pathogens) paired with well-defined endpoints such as multi-vessel coronary stenosis or first-ever myocardial infarction, effectively assessing non-identical exposure-outcome pairs. Second, existing MR approaches face inherent limitations of genetic instrumental variables: validated periodontitis-related SNPs capture only a small fraction of PD heritability, leading to weak instrument bias that reduces power to detect modest causal effects (Bell et al., 2020). Additionally, most MR datasets are derived from European ancestry groups, lack multi-ethnic validation, and limit generalizability to East Asian populations. Third, negative single-center case-control trials, such as the 2024 Iranian study, are limited by small sample sizes, which provide insufficient statistical power to detect moderate effect sizes of periodontitis on coronary atherosclerosis (Maboudi et al., 2024). Fourth, MR genetic proxies reflect lifelong inherited susceptibility to PD rather than the duration of actual inflammatory exposure; short-term mild periodontal inflammation may rarely drive atherosclerotic progression, whereas decades of untreated severe periodontitis induce persistent vascular damage, generating variable effect sizes across study populations. Fifth, the literature fails to distinguish epidemiological correlation from unidirectional direct causation. Consistent positive associations across observational and preclinical work support biological co-occurrence and plausibility, but do not prove PD acts as an independent primary trigger of CAD.

Collectively, these cross-design discrepancies do not negate the biological relevance of oral-cardiac interactions; they show that the PD-CAD relationship is context-dependent, shaped by population characteristics, disease severity, cumulative inflammatory exposure, analytical methodology, and variable operational definitions of exposure and outcome. This indicates that correlations identified by observational studies cannot be equated to causal relationships, and current evidence remains insufficient to establish a direct pathogenic effect of PD on CAD. Further verification through larger-sample, multi-ethnic MR analyses and long-term, large-scale randomized controlled interventional trials is required to disentangle correlational from causal pathways and validate sustained cardiovascular-protective benefits derived from standardized periodontal intervention.

## 4 Potential pathological mechanisms from PD to CAD

PD may drive the progression of CAD via synergistic, multi-layered molecular, cellular, and immune networks rather than a single, independent pathway (**Figure 1**).

**Figure 1:**
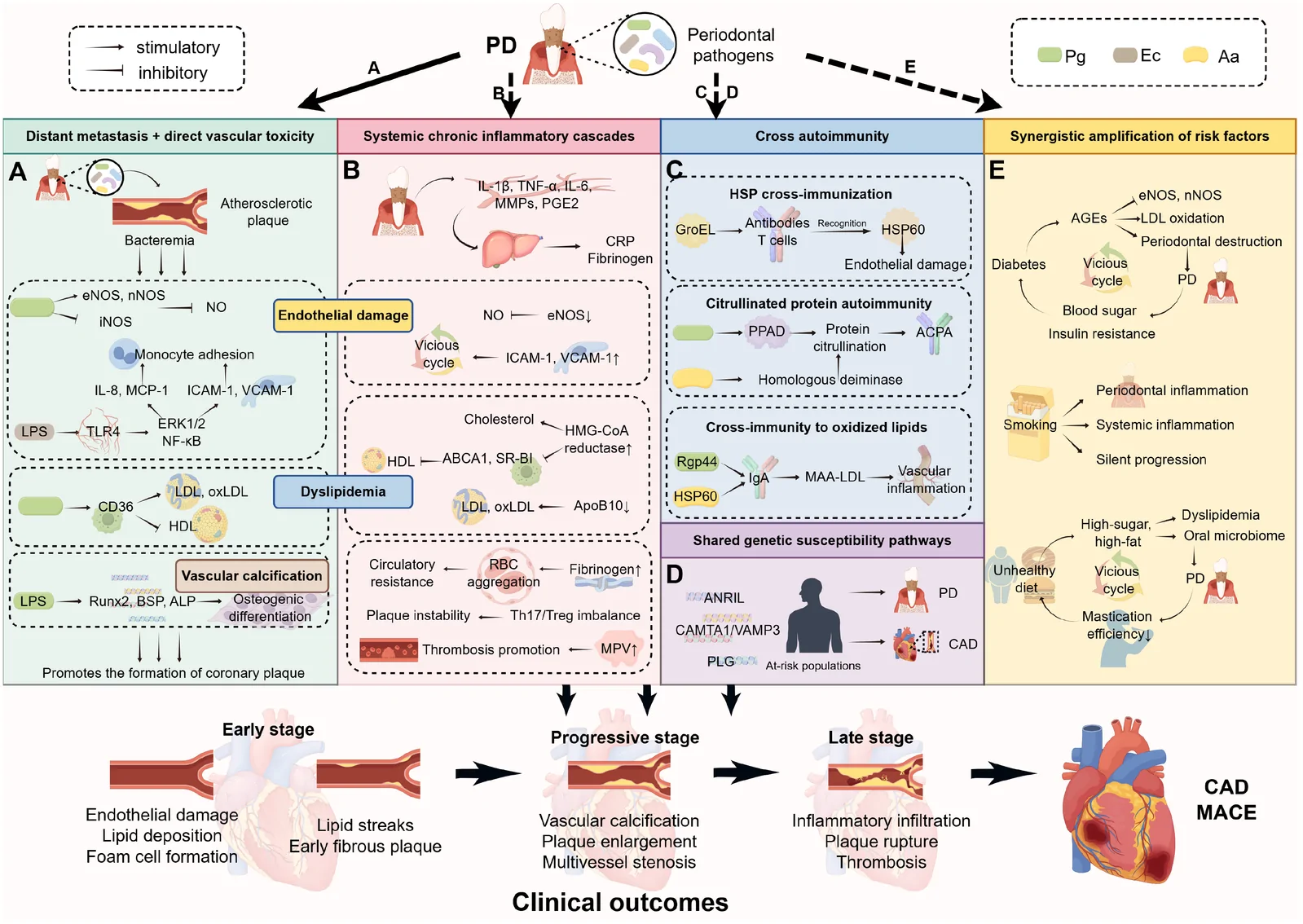
Schematic diagram illustrating the potential mechanisms linking PD to CAD. **(A)** Distant metastasis of periodontal pathogens and direct vascular toxicity, representing physical translocation of intact periodontal pathogens; **(B)** Systemic chronic inflammatory cascades; **(C)** Cross-autoimmunity; **(D)** Shared genetic susceptibility pathways; **(E)** Synergistic amplification induced by conventional risk factors. Pathways B-E are predominantly driven by virulence factors, antigens and inflammatory biological signals rather than translocation of whole bacterial cells. These interconnected pathways may collectively drive coronary atherosclerosis progression from early lipid-streak formation to plaque rupture, thrombosis and subsequent MACE. Abbreviation: Aa, Aggregatibacter actinomycetemcomitans; CAD, coronary artery disease; Ec, Eikenella corrodens; LPS, lipopolysaccharide; MACE, major adverse cardiovascular events; NO, nitric oxide; eNOS, endothelial nitric oxide synthase; nNOS, neuronal nitric oxide synthase; iNOS, inducible nitric oxide synthase; PD, periodontal disease; Pg, Porphyromonas gingivalis; oxLDL, oxidized low-density lipoprotein; HDL, high-density lipoprotein; ABCA1, ATP-binding cassette transporter A1; SR-BI, scavenger receptor BI.

### 4.1 Long-distance translocation, vascular colonization and direct vascular toxicity of periodontal pathogens

Dysbiosis of the periodontal microecology enriches subgingival biofilms with numerous Gram-negative anaerobic pathogenic bacteria, including core virulent strains such as Pg, Aa, Tannerella forsythia (Tf), Treponema denticola (Td), and Ec (Mahendra et al., 2015; Priyamvara et al., 2020). The thin, fragile sulcular epithelium can be disrupted during daily mastication, toothbrushing, periodontal probing, or dental procedures, allowing subgingival pathogens to invade the circulatory system and induce transient bacteremia, laying the groundwork for distant migration of bacteria to coronary vessel walls (Rydén et al., 2016; Priyamvara et al., 2020). PCR detection of clinical specimens from multiple studies has confirmed that homologous DNA of periodontal pathogens (predominantly Pg, Aa and Td) can be simultaneously identified in coronary atherosclerotic plaques and subgingival plaque samples harvested from the same patient (Pavlic et al., 2021; Mahendra et al., 2015, 2010; Ohki et al., 2012; Szulc et al., 2015; Rao et al., 2021). Directly verifying that periodontal pathogens can cross the circulatory system and colonize coronary atherosclerotic lesions.

Periodontal pathogens colonizing vascular walls and their virulence products (LPS, outer membrane vesicles, proteases, etc.) damage blood vessels and drive atherosclerosis through multiple independent mechanisms. Viafara-Garcia et al. demonstrated that Ec-derived LPS specifically binds the TLR4 receptor on human coronary artery endothelial cells (HCAECs), activating the ERK1/2 and NF-κB p65 signaling pathways (Viafara-Garcia et al., 2018). This triggers robust secretion of pro-inflammatory chemokines including IL-8 and MCP-1, alongside upregulation of ICAM-1 and VCAM-1, markedly enhancing monocyte-endothelial adhesion and recapitulating the pathological features of early endothelial injury in atherosclerosis (Viafara-Garcia et al., 2018; Higashi and Taguchi, 2025). Research using primary human aortic endothelial cells (pHAECs) by Sampath et al. revealed that Pg infection downregulates the endothelium-protective eNOS and nNOS and induces the expression of injury-inducible iNOS, thereby disrupting NO homeostasis and impairing vasodilatory function (Sampath et al., 2021).

Early animal experiments illustrated that repeated Pg bacteremia exposure significantly accelerates aortic and coronary atherosclerosis regardless of hypercholesterolemia status, indicating that periodontal pathogens exert independent pro-atherogenic toxicity (Brodala et al., 2005). Patients with PD typically exhibit an atherogenic lipid profile characterized by elevated LDL, triglycerides, small, dense low-density lipoprotein, very low-density lipoprotein, and oxidized LDL (oxLDL), alongside reduced cardioprotective high-density lipoprotein (HDL), thereby accelerating atherosclerotic plaque formation (Priyamvara et al., 2020). Elevated circulating oxLDL and LDL-derived particles further induce apoptosis of vascular smooth muscle cells (VSMCs), thereby exacerbating the risk of adverse cardiovascular events (Farrugia et al., 2021). Studies have found that Pg upregulates the LDL scavenger receptor CD36 on macrophages, accelerating intracellular oxLDL accumulation; simultaneously, it promotes HDL oxidation and blocks reverse cholesterol transport, thereby exacerbating lipid deposition in both directions and facilitating foam cell generation (Aleksijević et al., 2022). Furthermore, Pg-LPS stimulates abnormal proliferation of human umbilical artery vascular smooth muscle cells and upregulates key vascular calcification genes including Runx2, BSP and ALP, driving the transformation of smooth muscle cells into osteoblast-like cells and accelerating calcium nodule deposition in vascular walls, which increases the sclerosis of atherosclerotic plaques (Li et al., 2021).

Collectively, periodontal pathogens translocate across damaged periodontal epithelium into the bloodstream, enabling distant vascular colonization. Through multiple molecular pathways, they may directly disrupt the vascular endothelium, induce intracellular lipid accumulation, and initiate vascular calcification, thereby continuously promoting the initiation and progression of coronary atherosclerosis originating from local oral lesions.

### 4.2 Persistent systemic low-grade inflammatory cascade mediating sustained vascular endothelial injury

As a persistent, chronic, infectious, inflammatory disorder, PD triggers the massive release of pro-inflammatory cytokines, including IL-1β, TNF-α, and IL-6, from local immune cells in periodontal tissues in response to continuous stimulation by subgingival pathogens (Kumar et al., 2025). Mechanical stimuli such as daily mastication and toothbrushing continuously release bacterial virulence products and local inflammatory mediators into peripheral circulation, elevating serum levels of IL-1β, IL-6, TNF-α, matrix metalloproteinases, prostaglandin E2, and interferon-γ (Kumar et al., 2025; Jeffcoat et al., 2014; Sumayin Ngamdu et al., 2022; Wożakowska-Kapłon et al., 2013). Additionally, these substances stimulate hepatic synthesis of C-reactive protein (CRP) and fibrinogen, inducing systemic inflammation and vascular endothelial dysfunction (Sumayin Ngamdu et al., 2022; Cho et al., 2020).

Research has demonstrated that LPS-induced circulating elevations of IL-6 and TNF-α disturb lipid metabolism through dual mechanisms. On the one hand, they upregulate HMG-CoA reductase to boost endogenous cholesterol synthesis; on the other hand, they suppress the activity of the macrophage ATP-binding cassette transporter A1 and the scavenger receptor BI, thereby impairing HDL-mediated reverse cholesterol transport (Griffiths and Barbour, 2010). Moreover, LPS and outer membrane vesicles derived from pathogenic bacteria hydrolyze ApoB100, altering LDL structure, reducing its clearance efficiency and facilitating aggregation, thereby elevating circulating oxLDL levels and supplying lipid substrates for atherosclerotic plaque formation (Griffiths and Barbour, 2010). Systemic pro-inflammatory cytokines directly damage the vascular endothelium: TNF-α, IL-1β, and CRP synergistically upregulate endothelial surface ICAM-1, VCAM-1, promoting monocyte rolling, adhesion, and transmigration into the subendothelial space. This recruits additional inflammatory cells to secrete inflammatory mediators, forming a vicious cycle of “endothelial damage—inflammatory infiltration—aggravated endothelial injury” (Wojtkowska et al., 2021; Abou-Raya et al., 2007; Higashi and Taguchi, 2025). Elevated CRP further inhibits eNOS synthesis, downregulates the vascular-protective NO, and induces endothelial apoptosis, thereby further compromising vasodilatory function (Higashi et al., 2009; Hopkins et al., 2024). In addition, chronic inflammatory stimulation drives robust hepatic fibrinogen production, which promotes erythrocyte aggregation, increases blood viscosity, and elevates microcirculatory resistance; its predictive efficacy for cardiovascular risk even surpasses that of serum cholesterol (Wożakowska-Kapłon et al., 2013).

Beyond the above pro-atherogenic risk factors, colonization of periodontal pathogens within atherosclerotic plaques may exacerbate plaque instability and raise the risk of acute ischemic coronary events (Nocini et al., 2020). Periodontal pathogens may induce Th17/Treg immune imbalance, disrupting the equilibrium between pro-atherogenic and anti-atherogenic cytokines, intensifying inflammatory infiltration inside atherosclerotic plaques and increasing their instability (Aleksijević et al., 2022; Amato et al., 2023). Furthermore, studies have reported elevated mean platelet volume (MPV), enhanced platelet activation, and upregulated expression of thromboxane and adhesion molecules in patients co-affected by PD and CAD (Androsz-Kowalska et al., 2013). The risk of thrombosis surges following plaque rupture, ultimately triggering unstable angina, myocardial infarction, and other ACS.

### 4.3 Cross-autoimmunity mediated by molecular mimicry aggravating chronic vascular injury

Periodontal pathogens, may express antigenic epitopes that are highly homologous to those on human vascular endothelium and oxidized modified lipoproteins. Through molecular mimicry, these antigens break host immune tolerance and induce cross-reactive humoral and cellular immune responses that continuously attack autologous vascular tissues, aggravating chronic vascular immune injury and accelerating atherosclerosis progression (Liljestrand et al., 2018).

Cross-immunity against heat shock proteins (HSPs) represents the most classic pathway. Studies have found that periodontal pathogens produce the GroEL HSP, which is highly homologous to human HSP60. Long-term periodontal inflammatory stimulation induces the generation of antibodies and T cells that target GroEL, which cross-recognize autologous HSP60 on the vascular endothelium and continuously mediate endothelial immune injury, thereby disrupting vascular barrier function (Cho et al., 2020; Aarabi et al., 2018). The second major pathway involves citrullination of peptides triggering autoimmunity. Pg secretes peptidylarginine deiminase (PPAD), while Aa activates homologous human deiminases; together, these enzymes modify human fibrinogen, vimentin, and other proteins into citrullinated proteins. Sustained exposure to such antigens in genetically susceptible individuals may break immune tolerance to citrullinated proteins, generating anti-citrullinated peptide autoantibodies (ACPAs) that target citrullinated proteins within coronary plaques to amplify local inflammation, an effect particularly prominent in patients comorbid with rheumatoid arthritis (Aarabi et al., 2018).

Furthermore, cross-immunity related to oxidized lipids further amplifies vascular damage. Malondialdehyde (MDA), a lipid peroxidation product, forms immunogenic malondialdehyde-acetaldehyde (MAA) adducts that modify LDL to generate MAA-LDL autoantigens (Duryee et al., 2021). Two consecutive studies conducted by Akhi et al. identified homologous antigenic epitopes among Pg virulence factor Rgp44, Aa HSP60, and MAA-LDL. Mucosal IgA produced by the host against periodontal pathogens may cross-recognize vascular MAA-LDL, triggering autoimmune attacks and perpetuating chronic vascular wall inflammation (Akhi et al., 2019, 2022). Beyond inducing cross-autoimmune reactions, fimbriae on the surface of Pg may bind dendritic cell CD209 molecules, thereby helping pathogens evade immune clearance and sustain prolonged antigenic stimulation and persistent cross-autoimmune responses (Amato et al., 2023).

Cross-autoimmunity mediated by molecular mimicry constitutes an injury pathway independent of systemic inflammation, which may explain the clinical phenotype of sustained progression of coronary atherosclerosis in some patients without obvious elevation of systemic inflammatory markers. It supplements the immune regulatory mechanism by which PD may exacerbate chronic vascular damage.

### 4.4 Shared genetic susceptibility regulatory pathways

Genetic association studies indicate that PD and CAD share a panel of common genetic susceptibility loci. Innate genetic background may simultaneously elevate disease susceptibility to both disorders, forming the underlying molecular basis that regulates their comorbidity.

The CDKN2BAS (ANRIL) gene, located on the 9p21.3 chromosomal segment, is the first identified shared susceptibility gene (Schaefer et al., 2009). Functional variants at the rs10757278-G polymorphic locus within this region attenuate the binding affinity of transcription factor STAT, relieving STAT1-mediated transcriptional repression of CDKN2BAS and leading to aberrant overexpression of ANRIL, which simultaneously increases the genetic risk of PD and CAD (Shi et al., 2024). Variants at the CAMTA1/VAMP3 locus on the 1p36 region concurrently raise the risk of aggressive periodontitis and CAD (Bochenek et al., 2013). Additional research has identified the VAMP8-mediated membrane vesicle trafficking pathway as a shared molecular regulatory network for both disorders (Munz et al., 2018). PLG represents the third core shared susceptibility locus. Functional investigations have confirmed that all three shared genes are uniformly regulated by the TGF-β signaling pathway (Schaefer et al., 2015).

Genetic susceptibility pathways do not directly induce disease onset, yet they amplify vascular damage triggered by pathogenic bacterial infection, chronic inflammation, and autoimmunity. Carriers of risk genotypes exhibit higher vulnerability to coronary atherosclerosis under equivalent periodontal inflammatory burden, which may explain interindividual variation in the strength of the association between PD and CAD across populations.

### 4.5 Synergistic amplification effects of conventional risk factors

Traditional cardiovascular risk factors, including diabetes mellitus, smoking, and unhealthy diets, may exert bidirectional interactive effects with PD, capable of synergistically amplifying vascular damage mediated by all aforementioned pathways. These factors may serve as critical confounders that modulate the strength of the PD-CAD association.

Reciprocal interactions exist between diabetes and PD: diabetes impairs host immune resistance and exacerbates periodontal tissue destruction. In turn, PD continuously releases systemic inflammatory mediators that aggravate insulin resistance and hyperglycemia, thereby further accelerating atherosclerosis progression (Southerland et al., 2012). Chronic hyperglycemia in diabetic patients induces massive accumulation of advanced glycation end products (AGEs) within vascular walls. Intravascular AGE deposition inhibits NO production by eNOS and nNOS and promotes LDL oxidation, thereby aggravating periodontal tissue injury (Southerland et al., 2012; Khouja et al., 2019). Unhealthy lifestyles such as smoking weaken immune responses to infection, sustain chronic inflammation, and impair blood circulation (Zulkapli et al., 2026). Smoking also induces severe local periodontal inflammation and amplifies systemic inflammatory responses triggered by LPS (Rus et al., 2025; Khouja et al., 2019). Khouja et al. reported that PD does not significantly elevate CAD risk among non-smoking patients with type 1 diabetes; only when combined with smoking does PD become an independent predictor of CAD and severe coronary lesions, verifying the effect-amplifying role of smoking (Khouja et al., 2019). Furthermore, Tarnowski et al. reported that smoking may mask clinical manifestations of gingival microvascular lesions in diabetic patients: vasoconstriction induced by nicotine conceals hallmark periodontitis symptoms such as bleeding on probing, allowing silent progression of periodontal destruction (Tarnowski et al., 2018). The 2018 joint guidelines issued by the International Diabetes Federation (IDF) and European Federation of Periodontology (EFP) recommend comprehensive oral and periodontal examinations for all patients diagnosed with diabetes, with full-mouth periodontal pocket depth and bleeding index recordings completed following screening. Children and adolescents with diabetes require annual dental referrals for oral screening (Sanz et al., 2018).

A 2019 Brazilian cross-sectional study enrolling 115 patients with stable CAD found that individuals complicated by periodontitis consumed more sweets, legumes, and fried foods while consuming fewer fruits, failing to meet dietary standards for monounsaturated fatty acids (OR = 0.30, P = 0.04), and exhibiting significantly elevated serum triglyceride levels, which exacerbate lipid dysregulation. In contrast, patients retaining ≥20 natural teeth exhibited healthier dietary patterns (Mendonça et al., 2019). High-sugar and high-fat diets disrupt oral microecological balance and facilitate enrichment of periodontal pathogens (Betancur et al., 2025). Periodontal damage further reduces masticatory efficiency and limits intake of healthy food sources, creating a vicious cycle that may synergistically increase the burden of cardiovascular disease.

### 4.6 Section summary

Five non-exclusive, putative mechanistic pathways have been hypothesized to partly explain the potential link between PD and CAD, including distant translocation and presumed direct vascular toxicity of periodontal pathogens, cascades driven by persistent systemic low-grade inflammation, cross-autoimmune injury mediated by molecular mimicry, shared genetic susceptibility pathways, and synergistic amplification of vascular damage by conventional cardiovascular risk factors. *In vitro* cellular assays and limited human observational data provide partial, indirect support for each proposed route. Experimental findings suggest that periodontal pathogens and their virulence products may enter the systemic circulation, potentially causing endothelial injury, perturbing lipid profiles, and promoting vascular calcification. Sustained periodontal inflammation may elevate circulating pro-inflammatory mediators and thereby disturb vascular homeostasis. Antigen sequence homology between periodontal microorganisms and host vascular components raises the possibility of cross-reactive immune-mediated vascular damage. Genetic association studies identify shared susceptibility loci regulated by TGF-β signaling, which may confer concurrent predisposition to both disorders. Moreover, conventional risk factors such as diabetes, smoking and unhealthy diets may interact reciprocally with periodontal disease and potentially amplify vascular pathological processes.

Important caveats and unresolved inconsistencies surround these mechanistic inferences. Much of the supporting evidence comes from *in vitro* cell-based experiments, which cannot faithfully recapitulate the complex *in vivo* immune-metabolic microenvironment within human atherosclerotic plaques. Because these biological pathways are tightly interwoven and likely activated concurrently in comorbid patients, human studies currently cannot reliably quantify each putative pathway’s relative contribution to atherosclerosis progression. Shared genetic loci represent susceptibility risk modifiers rather than direct triggers of vascular lesions, and conventional cardiovascular risk factors may confound or potentiate signals attributed to periodontal-derived pathogenic stimuli.

Beyond these pathways and confounders, another mechanistic scenario merits consideration: long-standing severe PD may act as a phenotypic marker reflecting an intrinsic host predisposition to heightened systemic inflammatory tone. Under this framework, such inherent inflammatory susceptibility, rather than PD-specific pathogenic cascades, could independently promote both periodontal tissue destruction and atherosclerotic vascular injury. This interpretation is conceptually separate from confounding induced by lifestyle exposures and shared genetic susceptibility loci, offering another plausible explanation for the observed epidemiological correlation between PD and CAD.

Taken together, available mechanistic evidence lends biological plausibility to the hypothesized PD-CAD crosstalk, yet definitive human-based validation for the clinical magnitude and independent role of each proposed pathway remains lacking.

## 5 Clinical management implications for patients with comorbid PD and CAD

Consistent epidemiological evidence and multidimensional pathological mechanisms have established a potential association between PD and CAD. Based on the above population-level evidence and molecular mechanisms, this section elaborates on three core dimensions: the value of periodontal indicators for cardiovascular risk stratification, a bidirectional, integrated, collaborative intervention for oral and cardiovascular disorders, and the construction of a multidisciplinary collaborative diagnosis and treatment model. It extracts practical comorbidity management strategies applicable to cardiology and stomatology clinical departments, providing actionable references for the joint prevention and control of the two chronic diseases.

### 5.1 Periodontal status as an biomarker for adverse prognosis of CAD

Multiple cross-sectional, coronary angiography, and long-term follow-up cohort studies unanimously confirm that quantitative clinical periodontal indicators can independently predict the severity of coronary stenosis and the risk of long-term major adverse cardiac events (MACE), and can be incorporated into cardiovascular risk stratification to assist in prognostic assessment. Coronary angiography-related studies demonstrate that PPD and plaque index (PLI) are independently and positively correlated with the severity of coronary stenosis (Yang et al., 2013). Each 1 mm increment in PPD raises the odds of CAD by 25%, and higher staging of periodontitis corresponds to an increasing proportion of severe multivessel coronary stenosis (Lu and Lu, 2026; Nyagolova et al., 2024). Tooth loss, an irreversible terminal marker of periodontal tissue destruction, also possesses predictive value: loss of ≥10 natural teeth significantly elevates the risk of obstructive CAD, while loss of more than 20 teeth serves as an independent predictor of multivessel CAD (Lee et al., 2019; Cinezan et al., 2026). Further case-control studies focusing on AMI populations indicate that the API and BOP can help predict the risk of acute coronary events, with rising API levels paralleling a higher incidence of myocardial infarction (Wojtkowska et al., 2021). Periodontal indicators may assist in reflecting the severity of myocardial injury.

Beyond routine clinical oral indicators, oral microbial biomarkers enable more refined long-term risk stratification. Longitudinal follow-up cohorts after coronary artery bypass grafting show that colonization abundance of subgingival Campylobacter spp. independently predicts long-term adverse cardiovascular events in patients undergoing bypass surgery (Schulz et al., 2022). Quantitative peripheral blood nucleic acid detection of Pg is significantly correlated with the onset of AMI and the complexity of coronary lesions (Wu et al., 2023). Detection of oral microorganisms is expected to become a novel auxiliary tool for risk stratification of high-risk CAD populations. Overall, periodontal-related indicators are easy to collect, non-invasive and widely accessible, suitable for routine screening in cardiovascular outpatient clinics, and provide low-cost risk assessment tools for primary and secondary prevention of CAD.

### 5.2 Bidirectional collaborative intervention strategies

Standardized periodontal intervention can reduce the systemic inflammatory burden and improve vascular endothelial function, while basic cardiovascular medications and dietary nutrition regulation can, in turn, alleviate periodontal inflammation. Synchronized intervention of both conditions achieves dual benefits for oral and cardiovascular health, forming a bidirectional collaborative intervention scheme for comorbid patients.

Non-surgical periodontal therapy (NSPT) represents the core first-line intervention for periodontitis, consisting of supragingival scaling, subgingival curettage and root planing. Its core function is to eliminate pathogenic biofilm on root surfaces, reduce the total load of periodontal pathogens, and block the spread of bacteria and their toxic products into blood circulation through damaged periodontal epithelium (Jeffcoat et al., 2014). As early as 2006, Elter et al. found that treatment of periodontal infection can lower systemic inflammatory markers and improve vascular endothelial function (Elter et al., 2006). Meta-analyses confirm that NSPT significantly reduces serum levels of pro-inflammatory factors, including CRP, TNF-α, and IL-6, in CAD patients with periodontitis, and simultaneously improves fasting blood glucose levels (Su et al., 2025). However, this anti-inflammatory and endothelium-protective effect is time-limited, with ameliorative effects gradually attenuating within 6 months of the intervention. Clinically, patients with combined CAD and periodontitis are recommended to receive periodontal maintenance follow-up 2–3 times annually to sustain long-term benefits (Su et al., 2025). Studies confirm that mechanical debridement via NSPT is safe for CAD patients taking long-term low-dose aspirin, with reliable local hemostasis achievable without drug interruption (Ding et al., 2017).

Conventional cardiovascular drugs also exert periodontal-protective effects. Statins simultaneously inhibit local inflammation in blood vessels and periodontal tissues, delivering dual benefits for cardiac and oral health (Nocini et al., 2020; Alkakhan et al., 2023). Omega-3 (Ω-3) fatty acids downregulate the expression of periodontal matrix metalloproteinases, inhibit alveolar bone resorption, reduce systemic inflammatory markers such as the neutrophil-to-lymphocyte ratio (NLR), and exert local oral anti-inflammatory effects while potentially lowering the risk of cardiovascular events (González-Alva et al., 2024; Ahmad et al., 2026). Such nutrients demonstrate good safety in comorbid patients receiving long-term statin and aspirin therapy. Yet, their long-term protective effects on the prognosis of both diseases require further verification through large-sample, long-term interventional trials (Ahmad et al., 2026).

In addition, research identifies sphingomyelin as a shared metabolic molecule for PD and atherosclerotic cardiovascular disease (ASCVD), implying overlapping metabolic pathways between the two conditions (Bezamat et al., 2025). Synchronous monitoring of oral and peripheral blood metabolic biomarkers is expected to identify new targets for individualized, precise interventions and to optimize prevention and treatment regimens for patients with comorbidities.

Nevertheless, poor adherence to comorbidity intervention is prevalent in clinical practice. CAD patients complicated with PD show low adherence to standardized long-term cardiovascular treatment, which substantially elevates the risk of long-term adverse cardiovascular events (Rus et al., 2025). This phenomenon arises partly from insufficient patient awareness of the intrinsic correlation between the two diseases and partly from the traditional, fragmented diagnostic and treatment system that separates stomatology and cardiology. Cardiologists often overlook periodontal health screening, while dental practitioners rarely deliver cardiovascular risk education, missing the optimal window for early combined intervention (Bawankar et al., 2021). To address this status quo, clinicians should transform diagnostic thinking and establish cross-disciplinary collaborative intervention logic. Lee et al. recommend regular oral health assessment and periodontal health education for cardiovascular high-risk individuals under 65 years old (Lee and Hwang, 2021) Cardiologists should routinely inquire about periodontal symptoms such as gingival bleeding, tooth mobility, persistent halitosis, and tooth loss during outpatient consultations, and promptly refer patients with suspected periodontitis to the stomatology department for systematic periodontal treatment, constructing an integrated cardiac-oral prevention and treatment model (Bertoldi et al., 2024).

### 5.3 Multidisciplinary collaborative management track

The global prevalence of PD remains extremely high. Incorporating periodontal screening into routine cardiovascular chronic disease management can significantly reduce the societal burden of cardiovascular diseases, even if periodontal intervention only moderately lowers population-wide cardiovascular event risk (Lu and Lu, 2026). This highlights the public health value of a multidisciplinary collaborative model that integrates cardiology and stomatology.

Thus, clinics may establish a bidirectional screening mechanism: cardiology departments integrate inquiries about periodontal symptoms and brief periodontal examinations into initial cardiovascular consultations, with priority given to complete full-mouth periodontal assessment for high-risk populations including patients with hypertension, diabetes, dyslipidemia, prior myocardial infarction, or PCI status. Meanwhile, stomatology departments collect comprehensive cardiovascular medical histories during consultations and recommend timely cardiovascular disease screening for patients with multiple cardiovascular risk factors or severe periodontitis, thereby enabling early identification and intervention for both conditions.

It is also essential to disseminate oral health knowledge to the general public. Popularizing the link between chronic periodontal inflammation and coronary lesions is critical for preventing both oral and cardiovascular diseases (Etta et al., 2023). For populations with high comorbidity rates such as diabetics, smokers, and elderly individuals, integrated prevention and control strategies including standardized oral cleaning, regular periodontal maintenance, smoking cessation, and glycemic control should be promoted to manage shared risk factors of the two diseases synchronously.

## 6 Discussion

Cumulative evidence from cross-sectional, case-control, prospective cohort, and interventional follow-up studies consistently supports a graded positive correlation between periodontal inflammatory burden, pathogen colonization, and multiple adverse cardiovascular outcomes, including elevated CAD incidence, aggravated coronary stenosis, and increased long-term risk of major adverse cardiac events. Microbial testing of human atherosclerotic lesions further provides direct biological evidence that core periodontal pathogens such as Pg and Aa can translocate through damaged periodontal epithelium into systemic circulation and colonize vascular plaques, laying a biological foundation for the oral-cardiac interaction summarized in this review. *In vitro* cellular experiments and genetic association analyses further delineate five mutually synergistic pathogenic cascades underlying this comorbidity axis: distant vascular colonization and direct endothelial toxicity of periodontal pathogens, sustained systemic low-grade inflammatory responses disrupting vascular homeostasis, cross-autoimmune injury induced by molecular mimicry, shared genetic susceptibility loci modulated by TGF-β signaling, and synergistic amplification of vascular damage driven by traditional cardiovascular risk factors including diabetes, smoking and unhealthy dietary patterns. From a translational perspective, routine periodontal clinical indicators and oral microbial biomarkers serve as accessible, non-invasive tools for cardiovascular risk stratification. In contrast, standardized non-surgical periodontal therapy can reduce systemic inflammatory load and restore endothelial vasodilatory function, underscoring the need for bidirectional collaborative screening and multidisciplinary cardiostomatological management for patients co-affected by PD and CAD.

Despite the consistent positive correlation supported by observational and preclinical data, substantial discrepancies exist across the published literature, as reflected in null causal findings from MR analyses and a small subset of single-center case-control studies. These conflicting outcomes do not negate the biological relevance of oral-cardiac crosstalk but instead stem from systematic heterogeneity in study design, analytical methods, variable operational definitions for PD exposure and CAD outcomes across primary studies, population characteristics, and disease assessment criteria. Observational studies quantify epidemiological correlations and cannot fully eliminate residual confounding from overlapping lifestyle and metabolic risk factors, potentially leading to an artificial overestimation of PD’s independent effect on atherosclerosis progression. In contrast, MR approaches use germline genetic variants as instrumental variables to mitigate reverse causality and confounding bias. Yet, they are inherently limited by weak-instrument bias, unavoidable genetic pleiotropy, and the overrepresentation of European-ancestry populations, which restricts generalizability to multi-ethnic cohorts. Small negative clinical trials are further constrained by insufficient sample size and overly strict diagnostic thresholds limited to severe periodontitis, failing to capture the moderate vascular injury effects induced by decades-long mild-to-moderate periodontal inflammation. Notably, interpretation of this body of evidence must account for distinct inferential capacity across study designs: observational data establish correlation or temporal sequence rather than causality; *in-vitro* experiments only support biological plausibility; periodontal intervention trials demonstrate improvements in surrogate vascular-inflammatory endpoints among patients with established CAD but cannot test whether PD initiates de-novo coronary atherosclerosis.

Notable translational gaps remain between mechanistic research and real-world clinical practice, limiting the adoption of integrated cardio-stomatological interventions. Most existing periodontal intervention trials assess only short-term surrogate markers, such as circulating inflammatory cytokines and endothelial function, and lack long-term follow-up data on hard cardiovascular endpoints, including myocardial infarction, cardiovascular death, and repeated revascularization. Current genetic causal evidence is predominantly from European populations, and multi-ethnic genome-wide association and MR analyses are still needed to validate race-specific shared susceptibility loci and reduce population bias in causal inference. In addition, disciplinary segmentation between cardiology and stomatology persists in routine clinical systems, with no standardized bidirectional screening protocols for high-risk populations; insufficient public awareness of the oral-cardiac connection also leads to poor long-term treatment adherence among patients with comorbidities. To address these unresolved controversies and translational limitations, future research could prioritize large-sample, multi-ethnic long-term randomized controlled trials to quantify the sustained cardiovascular benefits of standardized periodontal maintenance, multi-omics integrated analyses to identify novel shared predictive biomarkers for PD-CAD comorbidity, and multicenter clinical implementation studies to develop feasible multidisciplinary collaborative diagnosis and treatment workflows suitable for routine chronic disease management. Collectively, the triangulated evidence synthesized in this review confirms the potential interplay between PD and CAD, while suggesting the need for further high-quality, multidimensional research to fully disentangle correlational from causal relationships and optimize population-wide joint prevention strategies for these two prevalent chronic disorders.

## 7 Conclusion

Existing studies demonstrate positive correlations between PD and CAD, while MR cannot confirm direct causality. PD may drive atherosclerosis via pathogen translocation, systemic inflammation, autoimmunity, and shared genetic susceptibility. Periodontal indicators show potential value for cardiovascular risk stratification, and standardized periodontal intervention might ameliorate vascular injury. Multidisciplinary cardiac-oral collaborative management warrants promotion, yet further large-scale trials are needed to validate the causal link and long-term clinical benefits.
